# Emotion Regulation Flexibility in Adolescents: A Systematic Review from Conceptualization to Methodology

**DOI:** 10.1007/s10567-024-00483-6

**Published:** 2024-07-14

**Authors:** Ann-Christin Haag, Rohini Bagrodia, George A. Bonanno

**Affiliations:** 1 Department of Counseling and Clinical Psychology, Teachers College, Columbia University, 525 West 120th Street, Box 102, New York, NY 10027 USA; 2 Department of Child and Adolescent Psychiatry/Psychotherapy, University of Ulm, Steinhövelstrasse 5, 89075 Ulm, Germany; 3 German Center for Mental Health, DZPG, Ulm, Germany

**Keywords:** Emotion regulation, Flexibility, Adolescents

## Abstract

Considerable attention has been devoted to the concept of flexible emotion regulation, which de-emphasizes the importance of any specific regulatory strategy in favor of the flexible deployment of strategies in response to specific situational challenges. The bulk of research in this area has been conducted on adult samples. Research on emotion regulation flexibility (ERF) in youth has been documented in only a limited number of studies and using various definitions. This systematic review aims to gather and summarize different conceptualizations and methodological approaches of adolescent ERF. We incorporate these findings into a general framework to understand ERF and its role in adolescents’ emotional, behavioral and social functioning. Adhering to the PRISMA guidelines, 11 studies were included in the review. While ERF has been defined in various and inconsistent ways, the included studies utilized conceptualizations from two overarching domains: the regulation of expressed emotion and the repertoire of emotion regulation strategies. Promising approaches and future directions will be highlighted.

## Introduction

Decades of research on adults and youth has linked skills in emotion regulation (ER) with psychological well-being (reviewed Aldao et al., 2010; Gross, 2008) and healthy development (Calkins, 1994; Cole et al., 2004). ER in youth has been substantially studied given its role in psychological adjustment (reviewed in Adrian et al., 2011; Zeman et al., 2006), and well-being or psychopathology that appear later in adulthood (reviewed in Mullin & Hinshaw, 2007). Lower levels of youth ER abilities have been associated with internalizing and externalizing problems both cross-sectionally and longitudinally (Calkins & Howse, 2004; Eisenberg et al., 2001; Mullin & Hinshaw, 2007; Rothbart et al., 1994; Rydell et al., 2003) and across clinical outcomes (reviewed in Villalta et al., 2018; Silk et al., 2003; Connelly et al., 2012). Although ER in adults has similarly been linked to various facets of healthy adjustment, research in this area has observed variation in strategy efficacy across different situational characteristics, such as degree of control (Troy et al., 2013) or emotional valence of the context (Kalokerinos et al., 2017). As a result, ER in adults has increasingly been couched within the framework of flexible self-regulation (Bonanno et al., 2004). Although the ability to flexibly regulate emotions across childhood and adolescence can have important implications for mental health, physical well-being, and even resilience (Bonanno, 2021), research on ER in children and adolescents has been slow to incorporate this development. There is a lack of knowledge of what constitutes emotion regulation flexibility (ERF) in adolescents and how this is associated with outcomes of emotional, behavioral and social functioning. This is mostly due to inconsistencies in how ERF has been defined and assessed in adolescents. Accordingly, this review aims to systematically assess the literature on ERF in adolescents to date, particularly on how it has been conceptualized and operationalized, in order to ultimately advance future research in this area.

### Emotion Regulation Flexibility in Adults

Research and theory have identified a large variety of ER strategies that individuals can use to modify or control their emotional experiences. Historically, these strategies have been categorized as either adaptive (i.e., reappraisal) or maladaptive strategies (i.e., suppression) (Aldao et al., 2010; Gross, 2015; Marroquín et al., 2017). As greater emphasis has been paid to the constantly changing contextual demands of one’s environment, current theory and research on ER have focused on the flexible use of strategies, rather than the adaptiveness of any specific strategy (Bonanno et al., 2004, 2007; Coifman & Bonanno, 2010; Kalokerinos et al., 2017). A burgeoning body of research has highlighted the importance of ERF, in particular the ability to accommodate and if needed modify strategy use in relation to the specific context in which they are applied (Aldao et al., 2015; Birk & Bonanno, 2016; Bonanno & Burton, 2013; Hollenstein, 2015; Kashdan & Rottenberg, 2010). As elaborated in a heuristic review by Bonanno and Burton (2013), regulatory flexibility can be conceptualized in three serially related yet functionally independent steps. Understood as the first step in regulatory flexibility, **context sensitivity,** involves the ability to evaluate contextual cues and demands of the stressor situation. Research has shown that greater context sensitivity, in particular the ability to identify the absence of threatening cues, is associated with fewer psychopathology symptoms (Bonanno et al., 2018). The subsequent step in this sequence, **repertoire,** involves the ability to access a wide range of strategies that may be implemented to meet such demands. Findings have shown better adjustment following stressful and potentially traumatic events is associated with use of a greater number of strategies (Orcutt et al., 2014), increased temporal variability (Cheng, 2001), and higher categorical variability (Chen et al., 2018). Finally, a third step, **feedback responsiveness,** involves the capacity to monitor the efficacy of a chosen strategy and modify or replace the strategy as needed. Research using real-time ecological momentary assessment (EMA) data has shown that the ability to discontinue situationally maladaptive strategies and switch to an alternative strategy is associated with fewer depressive symptoms (Chen et al., 2024). Other research has shown that certain strategies may be better suited to the situation depending on the controllability of the stressor or the intensity of physiological reactivity, where frequency of switching from reappraisal to distraction predicted better psychological adjustment (Birk & Bonanno, 2016).

ERF has been predictive of psychological adjustment in the aftermath of adverse events (Bonanno et al., 2004; Gupta & Bonanno, 2011; Rodin et al., 2017; Westphal et al., 2010) and lower levels of psychopathology symptoms (Moore et al., 2008; Zhu & Bonanno, 2017). Relatedly, use of distinct repertoires of ER strategies and the differential contextual application has been linked to well-being (Grommisch et al., 2020) and differentially associated with psychopathology profiles (Dixon-Gordon et al., 2015) and neurocognitive markers (Myruski et al., 2019).

### Development of Emotion Regulation Flexibility Processes Across Childhood Into Adolescence

Given that one of the most important tasks throughout childhood is learning to regulate emotions, there has been considerable interest in ER processes in youth (Zeman et al., 2006). Successful ER has been linked to positive adjustment through the lifespan and promotes social and psychological functioning (reviewed in Compas et al., 2017; Zeman et al., 2006). Developmental theories and reviews have also suggested that children and adolescents can demonstrate components of regulatory flexibility and learn to flexibly regulate their emotions by increasingly differentiating and matching the use of and appropriateness of ER strategies to various contexts as they age and build their strategy repertoire (Cole et al., 2004; Jones et al., 1998; Raffaelli et al., 2005; Sabatier et al., 2017; Skinner & Zimmer-Gembeck, 2007).

Much of the research on potential flexibility in youth ER has focused on early and middle childhood. For example, the selection between multiple ER strategies to appropriately regulate negative emotion has been documented in children as young as pre-schooled age (Dennis & Kelemen, 2009; Skinner & Zimmer-Gembeck, 2007) and predicted lower levels of behavior problems when preschool-aged children were reassessed at kindergarten age (Lunkenheimer et al., 2011). Similarly, preschool children who were able to report the use of a variety of ER strategies showed fewer hyperactivity and attentional problems as they grew older (Thomsen & Lessing, 2020).

In transitions from early to middle childhood, children have been shown to demonstrate rapid advances in the ER strategy repertoire, specifically in their knowledge, size, and effective use of increasingly more sophisticated ER strategies, including the expansion to cognitive-based strategies (Rice et al., 2007; Skinner & Zimmer-Gembeck, 2007; Thomsen & Lessing, 2020), and the ability to switch between strategies as needed (i.e., Parsafar et al., 2019). Children aged 5 to 6 (Davis et al., 2010) and 7 to 9 (Waters & Thompson, 2014) were able to demonstrate the differential selection of ER strategies to effectively regulate specific emotions elicited across various situations. Furthermore, children, aged 8 to 11 years old, were able to display context sensitivity, or the awareness that certain ER strategies may be more appropriate in some contexts than others (Quiñones-Camacho & Davis, 2020). Lastly, ER repertories and the potential varied use of strategies has been positively associated with children’s self-reported empathy and prosocial behavior (Gust et al., 2017). Similarly, children aged 10 to11 with attention-deficit/hyperactivity disorder (ADHD) reported fewer strategies overall and more antisocial strategies than same-age children without ADHD, highlighting the correlation between access to broader ER strategy repertoires and children’s healthy functioning (Babb et al., 2010).

Despite the promising findings on ERF in childhood, systematic research in adolescence is nonetheless lacking. This is both surprising and concerning as the appropriate regulation of emotion expression is widely viewed as an important milestone in both middle childhood and adolescence (Zeman et al., 2006). Adolescence is often marked by the experience of greater intensity of emotions and heightened emotional reactivity (Gullone & Taffe, 2012; Silk et al., 2003; Stifter & Augustine, 2019; Zeman et al., 2006). Furthermore, increased sensitivity to the peer evaluation and interpersonal consequences of particular displays of emotion renders the ability to flexibly modulate emotion expression as a crucial developmental skill (Wang et al., 2020). Many individuals respond to the milestones and challenges of adolescence by strengthening and refining ER skills, but for some, adolescence is marked by emergent or worsening difficulties with ER and associated psychopathology (Silvers, 2022). Finally, adolescence is typically a period where behavior becomes more flexible as a result of normative maturational processes, such as the onset of puberty. These abrupt changes and increased points of sensitivity are often referred to as phase transitions in the developmental timeline of an individual. During a phase transition, emotional systems can be reorganized and novel emotional patterns can emerge indicating increased ERF in adolescence (Granic, 2005).

### The Current Study

For the purposes of the present review, we focus on adolescent ERF abilities, i.e., the ability to flexibly use available ER strategies according to situational demands. We do not, however, include research on affective variability, which captures the degree of affective fluctuations. In this way, our review can be differentiated from previous literature that has highlighted and summarized developmental research on how affective states change over time and how these emotion dynamics impact psychosocial adjustment (reviewed in Hollenstein et al., 2013; McKone & Silk, 2022).

Given the fundamental role of ERF in emotional adjustment across the lifespan, in the current study we focus on a growing body of theory and research that identify measures of ERF in adolescence and their subsequent impact on emotional and behavioral functioning. A sizeable body of research in adults has linked ERF to positive effects on psychological adjustment in the aftermath of adverse events as well as lower levels of psychopathology symptoms in general (e.g., Gupta & Bonanno, 2011; Rodin et al., 2017; Zhu & Bonanno, 2017). However, considerably less is known about ERF in adolescents or its associations with psychological well-being. Both the conceptualization and the measurement of ERF in adolescence vary broadly. Consequently, there are not yet consensual conclusions about what constitutes ERF in adolescents and how it could serve as a potentially protective factor for adolescent functioning.

Addressing these gaps, the present study’s aims are twofold. First, this review seeks to identify and summarize studies that have evaluated the flexible use of ER strategies in adolescents, and we will present the existing conceptualizations and operationalizations of ERF in adolescents. Second, the review will describe associations of ERF with clinical outcomes, including emotional, behavioral and social functioning.

## Methods

A systematic review approach was chosen for the current study as the relevant literature utilized a variety of operationalizations of the ERF construct, thus preventing valid aggregation for statistical approaches, such as meta-analysis.

### Eligibility Criteria

The Preferred Reporting Items for Systematic Reviews and Meta-Analyses (PRISMA) guidelines were used to guide and determine study inclusion. Articles were included in the present review if the following criteria were met:

1. The mean age of the sample fell between 11 and 17 years of age. We excluded studies of younger children given the noticeable lack of research on the adolescent population. Furthermore, research indicates that there may be developmental differences in the strategies used (Parsafar et al., 2019; Skinner & Zimmer-Gembeck, 2007), and thus different methodologies to capture various processes as dictated by age.
2. Studies were included if they assessed the use of multiple ER strategies. Given the focus of ERF above and beyond an individual regulatory strategy, studies were excluded if they focused only on a specific strategy of ER.
3. Studies were included if they provided a definition or operationalization of ERF.
4. Studies were excluded if they focused on affective variability, i.e., emotional states and the degree of their momentary fluctuations, rather than the active use of strategies to regulate emotions.
5. Studies were excluded if biological/physiological correlates of ERF were measured as the primary focus of the study.
6. Studies were excluded if they involved experimental manipulation of ER capacity or skill in either clinical or non-clinical populations (e.g., intervention studies).
7. Studies were excluded if they assessed broader constructs of flexibility beyond ERF, such as psychological or cognitive flexibility. Studies that measured “affective flexibility” were evaluated for inclusion adequacy on a case-by-case basis.
8. Studies were excluded if they assessed constructs of flexibility on a dyad or systems-level. Instead, this review focuses on the systematic presentation of articles that describe individual-level processes.
9. Lastly, we limited the search to empirical, English-language articles published in peer-reviewed journals. Consistent with previous meta-analytic reviews about emotion regulation (e.g., Aldao et al., 2010; Compas et al., 2017), book chapters, non-peer-reviewed journal articles, review articles, commentaries, abstracts of conferences and congresses, case-reports and dissertations were not included. By limiting our review to studies published in peer-reviewed journals we increased the likelihood that studies would be of acceptable quality.

### Search Strategy

A thorough search of three databases (PsycInfo, Embase, and PubMed) was conducted in May of 2023. Search terms were selected by reviewing a collection of relevant articles and referencing the keywords used in the literature around emotional flexibility with adult samples. Of note, while the focus of this review is on adolescent samples, youth and children search terms were included to ensure the review of all articles that included the age range of 11 to 17-year-old participants. Lastly, several expert researchers on these topics advised before search terms were finalized. Table 1 details the search strategy used across the databases, and articles were filtered based on match to in title and abstract. In addition, we searched the reference lists of relevant studies for any additional papers.

**Table 1:** Search terms used in the systematic literature search

	“emotion* (dys)regulat*” OR “emotion* control*” OR “emotion* difficult*” OR “emotion* express*” OR “affect* (dys)regulat*” OR “affect* control*” OR “affect* difficult*” OR “affect* express*” OR “coping (dys)regulat*” OR “coping control*” OR “coping difficult*” OR “coping express*”
AND	“switch*” OR “flexibl*” OR "variability" OR "repertoire"
AND	“youth” OR “child*” OR “adolescen*”

Databases: PsycInfo, Embase, PubMed; limiters: peer-reviewed, language: English, * = truncation

### Study Selection, Data Extraction and Risk of Bias Assessment

Each title and abstract were independently reviewed by two of the authors (RB, AH) using the Covidence Systematic Review Software (2023). If either primary reviewer of the studies rated it as of potential relevance, the full text article was retrieved. The same two reviewers independently assessed the full texts against the eligibility criteria. Any discrepancies were resolved through mediation of an independent third reviewer (GB). The two primary reviewers independently extracted data from the retrieved studies. Data were extracted on the sample, study design, definition of ERF, measurement of ERF, and primary findings.

**Fig. 1:**
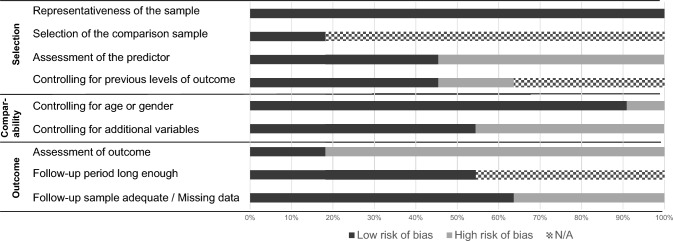
Risk of bias by domain and question across the included studies using the Newcastle–Ottawa Scale

The extent to which conclusions about ERF were drawn for the present review depended on whether data and results from the included studies are reliable and valid. Therefore, to evaluate the quality of the included studies, we used *the Newcastle–Ottawa Scale (NOS)*—a risk of bias assessment tool used for both case–control and longitudinal studies (Margulis et al., 2014). Studies are rated along three parameters (selection, comparability, and outcome) divided across eight specific items. Each item on the scale is scored with one point, except for comparability, which can be adapted to the specific topic of interest to score up to two points. While nine is the maximum number of points to achieve, less than 5 points indicate studies being at high risk of bias. Regarding the section “selection: outcome of interest not present at start”, we chose to consider this given if a study controlled for previous levels of the outcome in their analyses. “NAs” were given for studies with cross-sectional research designs. Similarly, for cross-sectional studies, “NA” was chosen for “Outcome: Follow-up long enough”. Lastly, for “Outcome: Adequacy of follow-up of cohorts” was answered given the authors management of missing data. A summary of the results is presented in Fig. 1. The three largest areas of concern were (1) the assessment of outcome variables, with most studies using self-report instead of a more objective measure, (2) the assessment of the predictor, with the majority of studies relying on self-report, and (3) the inclusion of additional control variables (except for age or gender). Handling of missing data also showed elevated risks of bias. Results should be interpreted considering these limitations regarding the quality of the included studies.

## Results

### Included Articles

**Fig. 2:**
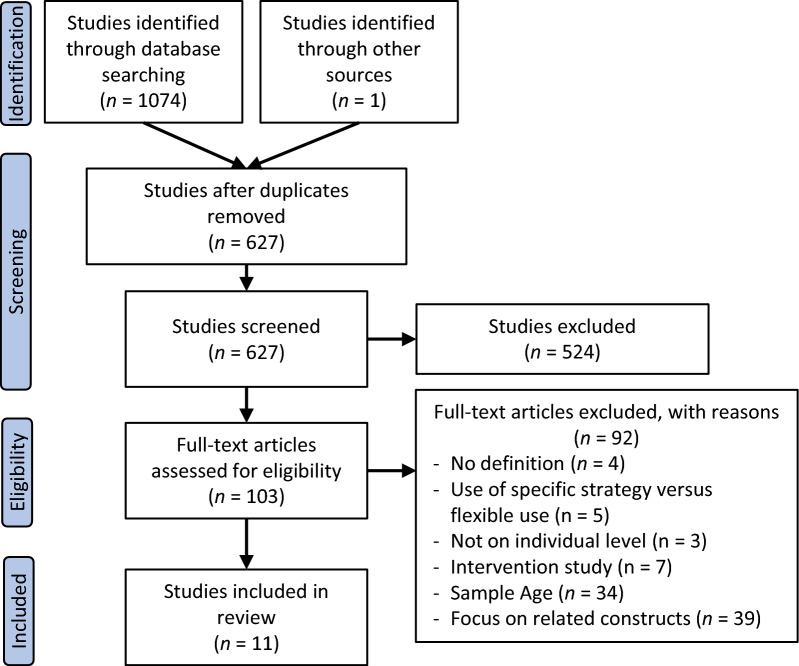
PRISMA flow chart for determining study inclusion

The initial search yielded 1,074 articles: 506 from PsycInfo, 294 from Embase and 274 from PubMed. One study was identified via forward citation searching (see Fig. 2 for the PRISMA flow diagram). After the removal of duplicates, 627 articles remained and were screened by title and abstract. From these 524 were excluded (135 due to non-relevance to scope or ER or ERF, 38 were not peer-reviewed empirical articles, 22 examined broader constructs of flexibility outside the scope of the present study, 143 were outside our of our desired age range, 59 examined biological proxy measures of ER, 51 examined the ER role and use as a features in clinical presentation, 56 evaluated ER as an outcome measure or within the scope of an intervention study, 11 studied parental influences on child or adolescent ER/ERF, and 9 examined specific ER strategies versus the flexible use of strategies). The remaining 103 studies were reviewed in full text. Of these, a following 92 were removed as these articles were found to not include a definition of ERF (*n* = 4), explored ERF  in a dyadic or family system, and not on an individual level (n = 3), examined the use of a specific strategy versus the flexible use of strategies (*n* = 5), included ER or ERF as an outcome or part of a larger intervention (*n* = 7), focused on related constructs (e.g., mood variability, psychological flexibility) rather than the flexible use of ER (*n* = 39), or the sample mean was revealed to be younger or older, and therefore did not satisfy our inclusion criteria (*n* = 34). Based on the inclusion criteria detailed above, a final 11 articles were included in this systematic review. Table 2 presents the summary of included studies and relevant study characteristics.

**Table 2:** Summary of included studies

Authors / Year	Location	Sample Size	Age Mean (SD)	Age Range (years)	Study Design	Definition ERF	Measurement of ERF	Primary Findings
Haag et al. (2022)	USA	Study 1: 439; Study 2: 172; Study 3: 43	Study 1: 11.42 (1.44); Study 2: 17.05 (1.97); Study 3: 13.11 (2.62)	8–19	Cross-Sectional study	Expressive flexibility: Ability to flexibly enhance or suppress emotional expression	Questionnaire (FREE-Y)	Validity of the FREE-Y; lower flexibility scores for maltreated versus comparison participants
Klosowka et al. (2020)	Belgium	214	NA	10–18	Cross-Sectional range of stressor types (IV); Adjustment outcomes (DV); ER role (Moderator)	Ratio of maladaptive/adaptive ER strategies to represent how ER operates in one participant; both the use and the adaptability of ER strategies depend on the specific context in which they are used	Questionnaire (FEEL-KJ)	Impact of stressors on psychological and physiological outcome moderated by flexibility scores
Lougheed and Hollenstein (2012)	Canada	177	13.6 (1.1)	12–16.9	Cross-Sectional study	ER repertoire that enables flexible deployment of multiple strategies (one at a time or multiple strategies at same time)	Questionnaires (ERQ, ASQ, DERS)	Latent profiles of five ER strategies. Greater ER repertoire associated with lower levels of internalizing problems
McKone et al., 2022	USA	129	12.26	11–13	Ecological momentary assessment (EMA)	Switching emotion regulation strategies	Self-report in EMA	Individuals differed in the extent of strategy switching. Switching strategies was associated with age & individual and within-person differences in perceived controllability, emotional intensity, and co-regulatory support
Mooney et al. (2017)	New Zealand	38	NA	5–15	Longitudinal/Descriptive phenomenological framework (interviews)/Qualitative coding	Varied use of multiple coping strategies to better manage diverse challenges	Interview coding	Greater coping abilities and post-disaster adjustment were associated with employment and use of diverse set of strategies
Schulz et al. (2005)	USA	72	14.6	14–15	Longitudinal Study	Modulation of emotional expression: the ability to modulate emotional expressions and responses	Interview coding	Better ability to modulate emotional expressions when negatively aroused was associated with lower hostility when parents displayed hostile behavior toward each other
Wang and Hawk (2019)	China	368	12.21 (1.58)	9–15	Longitudinal/ Experimental / Lab-based task	Expressive Flexibility: flexible modulation of emotion expressions to align with situational demands	Experimental Paradigm (adapted from adult EFT)	Expressive flexibility was associated with positive peer relations
Wang and Hawk (2020)	China	Study 1: 549; Study 2: 248, Study 3: 199; Study 4: 48	Study 1: 12.42 (1.70); Study 2: 12.74 (0.38); Study 3: 13.57 (0.63); Study 4: 12.43 (0.38)	8–16	Cross-Sectional/ Validation of Measure/ Experimental study	Expressive Flexibility: flexible modulation of emotion expressions to align with situational demands	Questionnaire (CAFE)	Validity of CAFE scale; lower flexibility scores were observed across clinical outcome variables (depression, problem behaviors)
Wang et al. (2020)	China	Study 1: 147; Study 2: 100	12.42 (0.38)	12–14	Cross-Sectional/Experimental Manipulation Paradigm	Expressive Flexibility: flexible modulation of emotion expressions to align with situational demands	Experimental Paradigm (adapted from EFT)	Bidirectional effects were observed between expressive flexibility and peer acceptance
Wang et al. (2022)	China	274	13.56 (0.63)	12–15	Longitudinal Study	Expressive flexibility: flexible modulation of emotion expressions to align with situational demands	Questionnaire (CAFE)	Friendship quality positively predicted expressive flexibility; expressive flexibility was a positive predictor for friendship quality for participants with lower social anxiety
Zimmer-Gembeck et al. (2018)	Australia	558	16.0 (1.50)	12–19	Cross-Sectional study	Access to a range of coping responses that can be flexibly deployed to match changing demands of stressful episodes	Questionnaire (SFCS)	Validity of the SFCS; lower scores were linked with greater anxiety and depressive symptoms, problem behaviors, and lower self-worth

*FREE-Y*  Flexible Regulation of Emotional Expression Scale – Youth, *FEEL-KJ* Fragebogen zur Erhebung der Emotionsregulation bei Kindern und Jugendlichen, *SPANE* Scale of Positive & Negative Experiences, *EFT* Expressive Flexibility Task, *CAFE* Child & Adolescent Flexible Expressiveness Scale, *SFCS* Self-Perception of Flexible Coping with Stress, *ERQ* Emotion Regulation Questionnaire, *ASQ* Affective Style Questionnaire, *DERS *Difficulties in Emotion Regulation Scale, *NA* not available

### General Study Characteristics

Studies were conducted in a wide variety of locales: Australia & New Zealand (*n* = 2), Belgium (*n* = 1), Canada (*n* = 1), China (*n* = 4), Netherlands (*n* = 1), and USA (*n* = 2). With respect to study design, a majority of the studies were cross-sectional (*n* = 7; 63%) and were conducted with a community sample (*n* = 9; 81%). Two studies were conducted including clinical samples: one study included a subsample of adolescents investigated for child maltreatment (Haag et al., 2022), and the other included a subsample of adolescents recently hospitalized for psychiatric difficulties (Schulz et al., 2005). Overall, data was gathered from 3,775 participants (sample size ranged from 17 to 1044 participants), with adolescents’ mean age being 13.39 years. Two studies did not report the mean ages (Klosowska et al., 2020; Mooney et al., 2017). To capture the developmental period of adolescence desired, one study was included where the age range spanned into children younger than 11 years (Haag et al., 2022) and two studies were included where the age range spanned into adolescents aged 19 years (Klosowska et al., 2020; Zimmer-Gembeck et al., 2018).

### Conceptualization of Emotion Regulation Flexibility

Across the included studies, ERF was conceptualized in a variety of ways. For the purposes of this review, we organized definitions into two categories: (1) regulation of expressed emotion and (2) repertoire of ER strategies. ERF was measured in four ways: Informant reports on questionnaires (*n* = 6), experimental paradigm (*n* = 2), as well as qualitative interview (*n* = 2), and EMA protocol (n = 1).

### Regulation of Expressed Emotion

Six studies operationalized ERF as the ability to flexibly modulate facial expressions of emotion in alignment with situational demands. One research group established the concept of *expressive flexibility* for youth, defined as the ability to flexibly enhance or suppress facial emotional expression in line with situational demands (Haag et al., 2022). Also adhering to this approach, four more studies defined ERF as *expressive flexibility*, assessed via either a laboratory task or a questionnaire (Wang & Hawk, 2019, 2020; Wang et al., 2020, 2022). One additional study defined ERF similarly and utilized the term *modulation of emotional expression* (Schulz et al., 2005).

Regulation of expressed emotion was captured through methods that spanned experimental paradigms (Wang & Hawk, 2019; Wang et al., 2020), questionnaires (Haag et al., 2022; Wang & Hawk, 2020; Wang et al., 2022), and interview (Schulz et al., 2005).

The experimental measure of *expressive flexibility* in the form of the *Expressive Flexibility Task* (EFT) was first developed and utilized with an adult population (Bonanno et al., 2004) and repeatedly validated across a wide variety of various community, veteran and clinical adult populations (i.e., Gupta & Bonanno, 2011; Rodin et al., 2017; Westphal et al., 2010). A version of the EFT was adapted for use with adolescents in China. This study followed the same instructions as the original task – while viewing emotion-inducing (positive and negative) pictures, adolescents were specifically instructed to either express their facial emotion expressions, suppress their emotional expression, or behave naturally. Blinded coders recorded the intensity of positive and negative emotion, and these codes were tabulated to create *expressive flexibility* scores following formulas defined in the original paradigm (Bonanno et al., 2004; Westphal et al., 2010). Expressive enhancement, expressive suppression, and an overall within-subject flexibility score were calculated (Wang & Hawk, 2019; Wang et al., 2020).

Relatedly, two questionnaires were developed to capture *expressive flexibility* in youth. The *Flexible Regulation of Emotional Expression Scale for Youth* (FREE-Y; Haag et al., 2022) was directly adapted from the adult version of the scale (Burton & Bonanno, 2016). Referring to Bonnano’s paradigm of *expressive flexibility* (Bonanno et al., 2004), researchers in China developed the *Child and Adolescent Flexible Expressiveness* (CAFE) scale (Wang & Hawk, 2020). Both self-report instruments are based on a scenario approach where adolescents rate their perceived ability to modulate (i.e., enhance and suppress) their displayed emotion across different hypothetical social scenarios. A flexibility score is derived from the enhancement and suppression scores.

Similarly, open-ended interviews of adolescents were transcribed and coded using the *Haan Q-sort of Defending and Coping Processes* (Haan, 1993). Of the 60 descriptors, eight captured an individual’s ability to modulate emotional and behavioral reactions, especially when challenged by difficulty or when experiencing distress. A higher score of modulation of emotions expression was indicative of greater ERF (Schulz et al., 2005).

### Repertoire of Emotion Regulation Strategies

Five studies defined ERF as the demonstration and varied use of a repertoire of diverse ER strategies (Klosowska et al., 2020; Lougheed & Hollenstein, 2012; McKone et al., 2022; Mooney et al., 2017; Zimmer-Gembeck et al., 2018). However, these studies slightly differ in their approaches. One study defined ERF as the ability to demonstrate the development of ER repertories and to determine at any particular moment whether the use of singular or multiple strategies was most needed (Lougheed & Hollenstein, 2012). Three other studies used this definition but expanded upon the role of context. Specifically, these studies defined ERF as the ability to demonstrate varied use of multiple strategies and successful determination of strategies that would be most adaptive across various contextual demands (Klosowska et al., 2020; McKone et al., 2022; Mooney et al., 2017). This definition highlights not only the variation in strategy use that may be most beneficial to an individual but also the necessity of evaluating context. Similarly, another study defined ERF as the ability to access a range of strategies that can be flexibly deployed to match changing demands of stressful situations (Zimmer-Gembeck et al., 2018).

In studies where ERF was based on a repertoire of ER strategies, the construct was measured via three methodological approaches: the use of questionnaires (*n* = 3), qualitative interview coding (*n* = 1), and EMA ( *n* = 1). The three studies utilized self-report questionnaires to measure adolescent ERF (Klosowska et al., 2020; Lougheed & Hollenstein, 2012; Zimmer-Gembeck et al., 2018). Klosowska et al. (2020) assessed ERF via the Dutch version of the German scale *Fragebogen zur Erhebung der Emotionsregulation bei Kindern und Jugendlichen* (FEEL-KJ; Cracco et al., 2015) to calculate the ratio of putative maladaptive ER strategy use to putative adaptive ER strategy use. Although the theoretical underpinnings of ERF eschew categorizing strategies as uniformly adaptive or maladaptive (referred to as *the fallacy of uniform efficacy*, Bonanno & Burton, 2013), the FEEL-KJ calculates both a total score for putatively adaptive and maladaptive strategies and a ratio of maladaptive ER strategy use over adaptive ER strategy use for each individual. Zimmer-Gembeck et al. (2018) developed a new measure, the *Self-Perception of Flexible Coping with Stress* (SFCS) scale that assesses the range of ER strategies and the extent to which they were flexibly deployed to match changing demands with three dimensions: multiple coping strategy use, situational coping, and coping rigidity. Multiple ER strategy use was measured as the extent to which an individual endorsed confidence in using multiple and new strategies; situational coping was represented by items that recorded the extent to which an individual understood the use of different ER strategies for different situations; and lastly, coping rigidity was measured by items that captured the extent to which an individual utilized the same ER strategy or demonstrated a lack of ability to change strategies. Mean dimensions scores were calculated as a representation of different components of ERF.

McKone and colleagues (2022) employed a daily life EMA design over the course of 16 days to capture ERF, here broadly defined as ER strategy switching between assessments. At each assessment, adolescents were asked to report on their most recent negative social interaction and choose one ER strategy from nine options. ER strategy switching was then operationalized as reporting a different ER strategy at that assessment compared to the previous assessment. Defined this way, ER strategy switching produced measurable individual differences that were moderated by multiple individual and contextual factors, including age, emotional intensity of the negative interpersonal situation, perceived controllability, and co-regulatory support available (McKone et al., 2022).

Lougheed and Hollenstein (2012) indirectly measured ERF by first assessing the endorsement of ER strategies through various self-report questionnaires. Five ER strategies were captured: Reappraisal and suppression strategies were assessed with the *Emotion Regulation Questionnaire* (ERQ; Gross & John, 2003); Concealing and Adjusting were assessed and captured by the respective subscales of the *Affective Style Questionnaire* (ASQ; Hofmann & Kashdan, 2010); and Emotional engagement was obtained as the index score across four subscales of the *Difficulties in Emotion Regulation Scale* (DERS; Gratz & Roemer, 2004). Using these data, ERF was then defined as the ability to use multiple strategies at the same time. The authors also observed different patterns of ERF based on Latent Profile Analysis.

One study identified a repertoire of ER strategies and their use through qualitative coding of interviews conducted with children and adolescents (Mooney et al., 2017). Systematic thematic analyses were conducted to identify initial categories which were then organized into meaningful clusters and resulted in the identification of six major themes/strategies: regulating felt emotions, problem-solving, positive appraisal and reframing, helping others, getting support, and moving forward. ERF was then measured by the indication of how many strategies were utilized and when (under what settings) the different strategies were employed effectively.

### Associations of ERF to Emotional, Behavioral and Social Functioning

Across all included studies, irrespective of operationalization and measurement, ERF was positively related to a range of outcomes of emotional, behavioral, and social functioning. ERF has been shown to be significantly linked to lower levels of adolescents’ internalizing problems (Lougheed & Hollenstein, 2012), depression and social anxiety (Wang et al., 2022; Zimmer-Gembeck et al., 2018), higher endorsements of positive affect and fewer problem behaviors (Wang & Hawk, 2020; Wang et al., 2022), and to greater abilities of adapting to challenges and coping after an adverse event (Mooney et al., 2017). Similarly, findings indicated that greater ERF resulted in better overall management of stressors and various challenges and that ERF was associated with greater general self-worth (Zimmer-Gembeck et al., 2018). Furthermore, lower ERF scores were revealed among youths exposed to maltreatment compared to a healthy control sample and small but significant negative correlations were shown between ERF and depression, social anxiety, and school avoidance (Haag et al., 2022). The EMA study that captured real-time ERF oversampled for risk of internalizing disorder by screening adolescents for dispositional fearfulness and shyness and found that higher dispositional shyness was associated with a lower likelihood of ER strategy switching (McKone et al., 2022). ERF was significantly associated with interpersonal relationship and functioning; in a longitudinal study, greater friendship quality at time 1 predicted greater ERF at time 2 (Wang & Hawk, 2019; Wang et al., 2022). In addition, greater peer acceptance was seen among adolescent participants when ERF was endorsed by their partner, suggesting that peer exclusion may be linked to impairments in the development of expressive regulation (Wang et al., 2020). Regarding parental relationships, adolescents who displayed higher ERF abilities were less likely to show increased hostility to parental figures (Schulz et al., 2005).

## Discussion

We sought to provide an overview of the various conceptualizations of the ERF construct as it applies to a younger population, and to evaluate the operationalization of the construct to date. We found that only a limited number of studies have investigated and defined adolescent ERF. In addition, the construct has been defined in various and inconsistent ways thereby challenging our understanding of what constitutes ERF in adolescents and how it could serve as a potentially protective factor for adolescent functioning. Our review identified 11 studies that utilized two overarching definitions of ERF in adolescents: (a) the regulation of expressed emotion and (b) repertoire of ER strategies. A majority of studies focused on the former definition of investigating individuals’ ability to flexibly modulate their emotional expression according to situational demands. This work is compatible with the recent shift in ER research in adults from individual ER strategies viewed as inherently adaptive or maladaptive towards a more person-situation perspective that emphasizes the match between strategy use and contextual demands (Bonanno et al., 2004).

### Conceptualizing Adolescent ERF

The studies we identified on youth ERF measured two isolated components, attending to situational demands and strategy repertoire. Theories of regulatory flexibility in adults typically encompass more elaborated multi-component ERF models. Bonanno and Burton’s (2013) model, described above, included three serially related component abilities, later termed the flexibility sequence (Bonanno, 2021): being sensitive to contextual cues, selecting from a diverse repertoire of ER strategies, and monitoring and potentially modifying an enacted strategy based on feedback regarding its efficacy. Not only does this model include additional components, e.g., use of corrective feedback, it also specifies the serial relationship of the components, e.g., the ability to decode the situational context feeds into the choice of strategy during the repertoire step, which in turn informs the action of the feedback step. Given that adolescence is characterized by considerable changes in cognition, emotion, and social relations, such multi-component serial models would be especially useful in guiding further research but also valuable to capture the dynamic nature of adolescence.

Regarding ER strategy repertoire, five studies included in the present review measured this dimension in terms of variability in ER strategy use across time and stressor situation, known as temporal variability (Bonanno & Burton, 2013). While temporal variability captures one aspect of repertoire, models of ERF have placed critical importance on the fit between strategy use and situational demands, i.e., strategy efficacy (Aldao et al., 2015; Bonanno et al., 2004, 2023; Levy-Gigi et al., 2016). The limitation of focusing only on strategy variability becomes readily apparent when its relationship to psychological adjustment is considered. Whereas conceptualizations of repertoire that take into account strategy efficacy are linearly related to adjustment (e.g., Bonanno et al., 2004, 2011; Cheng et al., 2012; Lenzo et al., 2021), strategy variability by itself has been shown to exhibit a curvilinear relationship to adjustment. More specifically, too much or too little strategy variability has been linked to psychopathology (Hollenstein et al., 2013; McKone & Silk, 2022).

Another crucial aspect of ERF not yet addressed in the developmental literature is the role of motivation. ERF is typically enacted in the context of emotionally evocative or distressing situations. Engaging with a stressor event to a sufficient degree to attend to its contextual nuances, enact a regulatory response, and then monitor its efficacy requires at least some cognitive and emotional resources (Bonanno et al., 2023). Although currently available theoretical models of ERF vary to some extent in their aim and scope, these models generally agree that engaging the resources required for ERF requires some degree of motivation (Aldao et al., 2015; Bonanno & Burton, 2013; Cheng et al., 2014; Kashdan & Rottenberg, 2010). At present, measurement of the motivational component in the adult ERF literature has been limited to self-report assessments. Nonetheless, it will be important for future research on ERF in youth to explore the role of motivation using these or other indices.

Lastly and importantly, future research will have to find greater consensus regarding the conceptualization of ERF in adolescents, to use consistent terminology and standardized measures in order to facilitate comparison across studies, underscore common patterns and derive the best practices to support adolescents.

### Related Frameworks in Developmental Psychology

While our review synthesizes the literature regarding adolescent self-regulation flexibility, it is worth considering the related concept of emotion dynamics, which has gained increased attention in recent developmental research. Emotion dynamics research focuses on analyzing how individuals’ emotional experiences, i.e., emotional states, change over time in response to context, and how this impacts psychosocial adjustment (reviewed in Hollenstein, et al., 2013; McKone & Silk, 2022). Two prominent emotion dynamics constructs have emerged, namely (1) *affective variability,* defined as reactivity to the interpersonal situation, with a focus on the rate of change in emotional states, and (2) *socioaffective flexibility*, defined as the ability to move through a range of affective states over time and a range of emotional states expressed during interpersonal interactions, usually measured in the context of caregiver–child dyad interactions. *Socioaffective flexibility* has also been referred to as *socioemotional flexibility* (Hollenstein et al., 2013) or *dyadic affective flexibility* (Mancini & Luebbe, 2016). These constructs reveal an interesting and often neglected aspect of ER, which is that ER not only manifests on the individual level, but also on interpersonal levels across systems. Dyadic approaches are important to consider as they measure two individuals’ ability to be flexible together across emotional reactions, can allow dyads to recognize interaction patterns and can create pathways to explore the influence of both individual characteristics (e.g., symptomatology) as well as dynamic family processes (Hollenstein et al., 2004; Mancini & Luebbe, 2021; Van Bommel et al., 2019). Dyadic or even triadic (Hollenstein et al., 2015) family-level approaches are also important as they provide insights into the impact of caregivers on their offspring’s emotion dynamics and ER development pertaining to conceptualizations of ER as a family-level phenomena (e.g., co-regulation; reviewed in Paley & Hajal, 2022).

A body of affective variability research has amassed that utilized different operationalizations to capture metrics of the construct, including the frequency of transitions among different emotion states and the duration of each emotion state, measured both on the individual and the dyadic level. While numerous studies used laboratory observational tasks to code expressed emotions (e.g., Van Bommel et al., 2018) and present them on state-space grids (e.g., Hollenstein et al., 2004, 2013; Mancini & Luebbe, 2021; Van der Giessen & Bögels, 2018; Van der Giessen et al., 2015), recent approaches also applied more intensive longitudinal data assessments using daily diary entries to assess daily emotion dynamics (Lichtwarck-Aschoff et al., 2009; Mak et al., 2023).

Both *affective variability* and *socioaffective flexibility* have been associated with internalizing and externalizing symptoms in children and adolescents, indicating that lower levels of (dyadic) affective variability are associated with greater levels of symptoms (e.g., Hollenstein et al., 2004; Lougheed & Hollenstein, 2016; Van der Giessen et al., 2015). However, the directionality of results has not been consistent across studies and associations between affective variability and outcomes of psychopathology were not always linear, but, as described before for temporal variability, at times curvilinear (McKone & Silk, 2022).

### Operationalization of Adolescent ERF

Another important area for future research will be to further probe the validity of ERF assessment approaches. The available studies used a broad range of measure to assess ERF, including questionnaires, observation, interview, diary coding and experiments. While such methodological variety has its advantages, the largest proportion of studies included in our review assessed ERF exclusively from self-report questionnaires (*n* = 6, 37.5%). More objective study designs including naturalistic settings are needed. One study used an EMA protocol, and only two studies used experimental approaches. To date the only existing experimental design to assess ERF is the EFT (Bonanno et al., 2004), which has been used almost exclusively with adult populations. While this paradigm was recently adapted for use with Chinese children and adolescents (Wang & Hawk, 2019), it has yet to be adapted and validated across diverse cultures and adolescent populations, including both clinical and community samples. A comparable self-report scale assessing ERF in youth, the FREE-Y (Haag et al., 2022) was recently adapted from an adult version. An advantage of this approach is its high level of external validity through its inclusion of real-life interpersonal scenarios. It will be critical, however, for future research to also validate the FREE-Y against more objective experimental data.

The various types of ERF assessments have different benefits. While experimental approaches maximize internal validity, they are limited in ecological validity due to their artificial nature and are limited in their potential for application to longitudinal or prospective field research (Burton & Bonanno, 2016). Self-report questionnaires, on the other hand, might represent a better way to capture subjective experiences and imply greater external validity. Observational coding of behaviors allows for an assessment of ERF abilities in naturalistic environments and thus can include detailed information about the actual context. In addition, daily diary or EMA study designs appear to be promising avenues for future research on ERF since they can offer a more thorough situational assessment.  Whereas one-shot measures of ER, such as questionnaires or experimental designs, have limited ability to capture important nuances in the ER process or how individuals use ER abilities across contexts, naturalistic designs provide informative data on how ERF abilities might influence social and emotional outcomes across different social contexts. Lastly, since ERF is conceptualized primarily as a process, capturing that process in real-time in reference to real-life challenges is crucial (Bonanno et al., 2023).

### Associations of ERF and Emotional, Behavioral and Social Functioning

In our review we also sought to elucidate the role of ERF in adolescents’ emotional, behavioral and social functioning. Despite their differing conceptualizations of adolescent ERF, the available studies demonstrated associations between ERF and more positive outcomes across an array of variables, including clinical outcomes, such as lower levels of depression and anxiety or fewer problem behaviors, and social outcomes, such as better relationship quality with caregivers and peers. While ERF research in adolescence is nascent, these results nonetheless attest to the potentially beneficial role of ERF supporting adolescent functioning and representing a protective skillset. These findings also suggest that deficits in ERF abilities, in turn, may serve as transdiagnostic mechanisms in the development and maintenance of difficulties in emotional, behavioral, and social functioning. Developmental psychologists have characterized “*mature* ER ability” as the ability to deploy various specific strategies in a manner that effectively matches changing situational demands (Skinner & Zimmer-Gembeck, 2007). Complimentarily, then, if ER abilities are not maturely developed, adolescents’ ability to flexibly regulate their emotions may be impeded and, in the extreme, may develop into full-blown psychopathology. Consistent with this supposition, one study in our review that included a clinical sample revealed lower levels of dyadic ERF in caregiver-child dyads where the child endorsed anxiety (Van der Giessen & Bögels, 2018). There is also evidence that ERF is reduced in adolescents exposed to maltreatment (Haag et al., 2022). It will hence be essential for future studies to investigate ERF in clinical adolescent samples (e.g., adolescents struggling with psychopathology and/or exposed to adverse events). This will increase our ability to generalize findings across various populations and complement findings from adult research by further establishing the buffering role of ERF (e.g., Bonanno et al., 2004; Westphal et al., 2010).

In terms of clinical implications, the corpus of research on ERF summarized in our review can inform intervention strategies to foster psychological adjustment and protect against untoward effects of potentially traumatic events. Training flexible self-regulation should be a target of intervention development research as it appears to be functioning as a transdiagnostic mechanism and could be applied in psychotherapeutic practice. Improving ERF skills could either be integrated into existing more global interventions, such as mindfulness-based stress reduction or acceptance commitment therapy, or a specific ERF training program that borrows elements from established approaches targeting related areas, such as Dialectical Behavior Therapy (Linehan, 2015). Given that the effectiveness of ER strategy use is dependent on fit with situational demands, flexibility interventions should aim to increase individual’s awareness and understanding of how they select, implement and, when needed, revise ER strategies.

### Limitations of the Current Review and Future Directions

Our review represents the first systematic effort to summarize existing conceptualizations of adolescent ERF to guide and unify future approaches. However, several important limitations should be considered. From a methodological point of view, our review was limited to studies published in English-language and in peer-reviewed journals in order to adjudicate quality. As such, there is a risk of reporting bias, possibly leading to relevant studies not being included in the present review. Further, the present review focused only on the existing literature in adolescents between the ages of 11 and 17 years. Future research needs to investigate age-related differences and developmental changes in ERF across childhood and adolescence and needs to take into account contextual factors. For example, during adolescence, sensitivity to social feedback increases (Somerville, 2013) as peer relationships become more important and less stable (Hardy et al., 2002), leaving adolescents vulnerable to experiences of victimization and rejection by peers. The use of social scenarios in assessment instruments, such as the FREE-Y (Haag et al., 2022), to some extent accommodates this sensitivity. However, additional research will be needed to more fully probe this point. It will also be crucial to address other key non-social aspects of adolescent ERF.

Finally, it will be important to expand ERF research beyond individual assessments to encompass the dyadic or family-level studies, as described above for affective variability. And, at a broader level, it will be crucial for future ERF research to probe different units of analyses, e.g., dyadic ERF at a caregiver-adolescent or family-system level, while also elucidating the relationship between adolescent and caregiver ERF. Such analyses would allow for examination of potential bidirectional effects, the role of ERF on a family level, as well as the intergenerational transmission of ERF abilities. In the same vein, it will be important to examine the role of socialization processes in the shaping of adolescent ERF.

## Conclusions

In the present review, we summarized 11 studies examining adolescent ERF from two broad perspectives: the regulation of expressed emotion and the repertoire of emotion regulation strategies. We highlighted the variety of conceptualizations and the range of applied methodologies. Future ERF research focusing on greater conceptual clarity, attention to research design and contextual demands can advance the understanding of ERF development and its impact on individual trajectories of psychological adjustment throughout the lifespan. More research is needed to extend the study of adolescent ERF by including the investigation of adolescents’ sensitivity to contextual demands, their use of feedback processes to adapt the use of ER strategies as well as motivational aspects of ERF in adolescents, if possible, within the same data set. It has become increasingly apparent that ERF plays an important role in adjustment to contextual challenges related to both development during adolescence in general as well in the context of highly stressful life events. For this reason alone, it is apparent that a more comprehensive study of ERF in youth is needed.
